# High genetic diversity at the regional scale and possible speciation in *Sebacina epigaea* and *S. incrustans*

**DOI:** 10.1186/1471-2148-13-102

**Published:** 2013-05-22

**Authors:** Kai Riess, Franz Oberwinkler, Robert Bauer, Sigisfredo Garnica

**Affiliations:** 1 Plant Evolutionary Ecology, Institute of Evolution and Ecology, University of Tübingen, Auf der Morgenstelle 1, 72076 Tübingen, Germany

**Keywords:** Cryptic species, Speciation, Diversity, Population structure, Multilocus genealogies, Basidiomycota, Sebacinales, Sympatry, Synonymous polymorphism, Ectomycorrhiza

## Abstract

**Background:**

Phylogenetic studies, particularly those based on rDNA sequences from plant roots and basidiomata, have revealed a strikingly high genetic diversity in the Sebacinales. However, the factors determining this genetic diversity at higher and lower taxonomic levels within this order are still unknown. In this study, we analysed patterns of genetic variation within two morphological species, *Sebacina epigaea* and *S. incrustans*, based on 340 DNA haplotype sequences of independent genetic markers from the nuclear (ITS + 5.8S + D1/D2, RPB2) and mitochondrial (ATP6) genomes for 98 population samples. By characterising the genetic population structure within these species, we provide insights into species boundaries and the possible factors responsible for genetic diversity at a regional geographic scale.

**Results:**

We found that recombination events are relatively common between natural populations within *Sebacina epigaea* and *S. incrustans*, and play a significant role in generating intraspecific genetic diversity. Furthermore, we also found that RPB2 and ATP6 genes display higher levels of intraspecific synonymous polymorphism. Phylogenetic and demographic analyses based on nuclear and mitochondrial loci revealed three distinct phylogenetic lineages within of each of the morphospecies *S. epigaea* and *S. incrustans*: one major and widely distributed lineage, and two geographically restricted lineages, respectively. We found almost no differential morphological or ecological characteristics that could be used to discriminate between these lineages.

**Conclusions:**

Our results suggest that recombination and negative selection have played significant roles in generating genetic diversity within these morphological species at small geographical scales*.* Concordance between gene genealogies identified lineages/cryptic species that have evolved independently for a relatively long period of time. These putative species were not associated with geographic provenance, geographic barrier, host preference or distinct phenotypic innovations.

## Background

Comparative studies of the genetic structure of populations, particularly those utilising molecular makers, have provided new opportunities for better understanding historical and/or contemporary factors for modelling speciation. Surveys using DNA variation in rDNA and other sequence data have revealed several examples of morphological fungus species that have diverged into reproductively and/or genetically isolated morphologically indistinguishable monophyletic subgroups or cryptic species see, e.g. [1-8]. In particular, patterns of geographic distribution have been noted as one of the main factors shaping species diversification. For instance, geographically isolated populations (allopatry) appear to be one of the most plausible causes of speciation. In addition, for some species complexes, sympatric speciation likely has been caused by changes in ecological [9] and/or life history traits and reinforced by negative selection towards hybrids. Such causes seem to be less frequent in allopatric speciation, where the major barrier to gene flow is geographic.

Gene genealogies from independent DNA loci have been recommended as a means of identifying phylogenetic species [6,10]. Molecular markers are very helpful for this purpose, especially within fungus groups with comparatively simple body plans that seriously restrict the number of constant and recognisable characters. This is the case in the Sebacinales, where high genetic diversity has been reported from the analysis of rDNA sequences of lineages with low levels of morphological variation, see e.g. [11]. For example, considerable intraspecific genetic variation has been found within the genus *Sebacina* (*S. epigaea*, *S. incrustans* and *S. vermifera*). While *Sebacina vermifera* is not known to develop visible basidiomata, *S. epigaea* develops relatively thin waxy-gelatinous, pale grey basidiomata [12], and *S. incrustans* forms thick resupinate-incrusting, coriaceous, cream to ochre basidiomata [13]. *Sebacina epigaea* and *S. incrustans* are both broadly distributed in Europe and form mycorrhizal associations with forest trees [14] and/or orchids [15]. Both species are relatively easy to recognise and distinguish from one another in the field providing an exceptional opportunity to study their intraspecific diversity in combination with an evaluation of their ecological and morphological characteristics.

**Figure 1:**
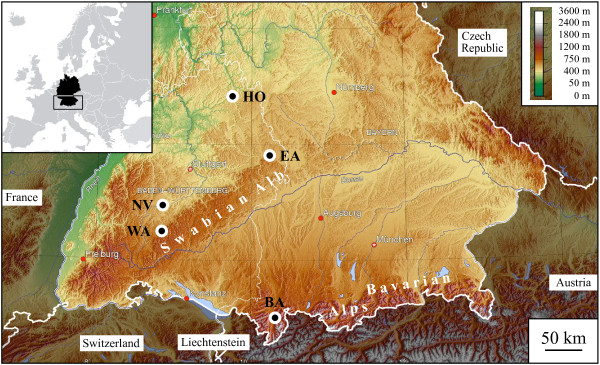
**Map of Southern Germany illustrating the geographic location of the collecting sites of *Sebacina epigaea *and *S. incrustans *population samples.** Sampling areas are coded as follows: BA = Bavarian Alps, EA = Eastern Swabian Alb, HO = Hohenlohe, NV = Neckar Valley, WA = Western Swabian Alb.

To explore the possible factors that have caused the high genetic diversity observed in the Sebacinales, we have focussed our molecular phylogenetic approach on population samples of two morphological species, *Sebacina epigaea* and *S. incrustans* distributed over a regional geographic range (Figure 1). By characterising the genetic variation, estimating the genetic structure, and comparing the independent nucDNA (ITS + 5.8S + D1/D2 and RPB2) and mtDNA (ATP6) markers from *S. epigaea* and *S. incrustans* population samples, we have been able to address the following questions: (i) What is the intraspecific genetic variation within these two morphospecies, how many species are found within each morphospecies and are these morphospecies monophyletic? (ii) Are their intraspecific nuclear and mitochondrial genealogies concordant? (iii) If significant genetic diversity is present, is there morphological, geographical and/or ecological segregation of the different major lineages?

## Results

### DNA amplification and phylogenetic placement

**Figure 2:**
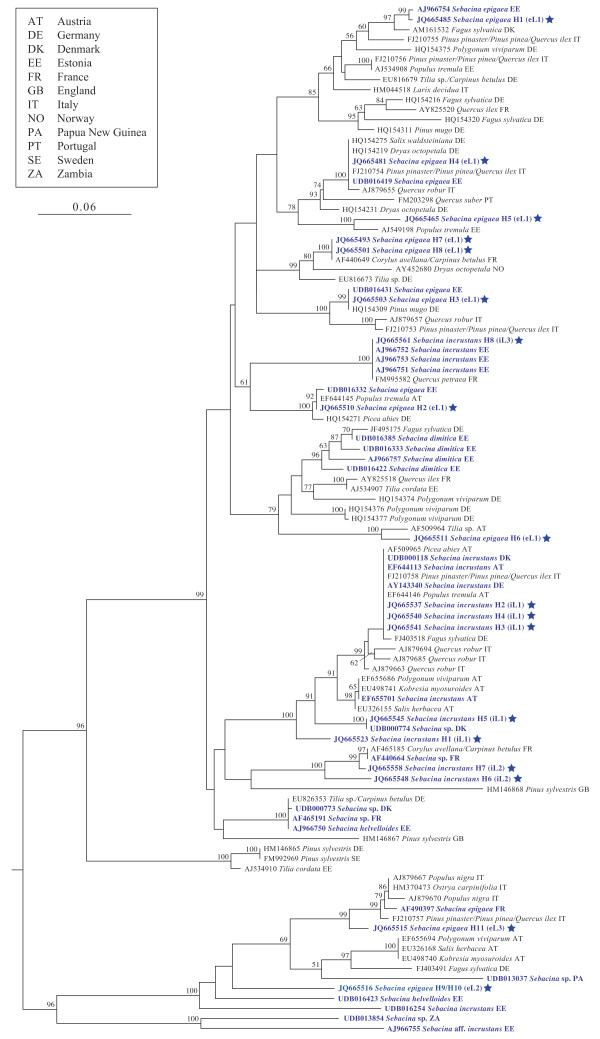
**Phylogenetic placement of the newly generated *Sebacina epigaea *and *S. incrustans *ITS rDNA sequences.** Ingroup includes our own *Sebacina* sequences (one per haplotype, marked with a star), those ITS sequences obtained from basidiomata of *Sebacina* (blue printed) and ectomycorrhizae of European trees available at GenBank and UNITE databases. Tree topology was computed from 1000 runs and was midpoint rooted. Bootstrap supports (>50%) are shown for each node. eL = *S*. *epigaea* lineage, H = haplotype, iL = *S*. *incrustans* lineage.

In general, it was easy to amplify the nuclear DNA (ITS + 5.8S + D1/D2 and RPB2 genes) from the majority of the population samples. On the other hand, for many population samples of *Sebacina epigaea,* we were unable to amplify the mitochondrial DNA (ATP6 gene). Phylogenetic analysis of our own complete ITS sequences and those of *Sebacina* basidiomata available from the GenBank/UNITE databases placed some of our sequences in rather isolated positions (Figure 2), except for *S. epigaea* haplotype H3 was identical to UDB016431 and haplotype H4 was identical to UDB016419 both from Estonia, respectively. For *S. incrustans,* haplotypes H2, H3 and H4 appeared to be identical to EF644113 (Austria), UDB000118 (Denmark) and AY143340 (Germany); haplotype H5 the same as UDB000774 (Denmark); and haplotype H8 to be identical to AJ966751, AJ966752 and AJ966753 (all Estonia).

In addition, some D1/D2 sequences of both species appeared to be identical to sequences of basidiomata in GenBank from Germany: *S. epigaea* H11 to AF291267; *S. incrustans* H1 to AF291365 and FJ644513; and *S. incrustans* H2, H3 and H4 to AY143340 and DQ520095, and from Austria: *S. epigaea* H2 to AY505560 (data not shown).

### Major phylogenetic lineages

**Figure 3:**
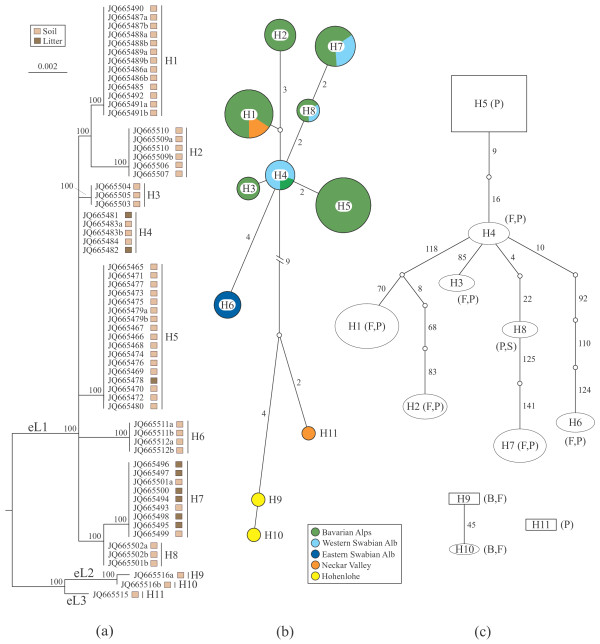
**Genetic variation based on 63 non-recombining ITS + 5.8S + D1/D2 haplotype sequences of *Sebacina epigaea*.** Haplotypes are coded with H1 to H11 and heterozygous sequences are coded with a and b. (**a**) Maximum likelihood phylogenetic tree. Tree topology was computed from 1000 runs and was midpoint rooted. Bootstrap supports (>50%) are shown for each node. Substrate types where the basidiomata grew are mapped on the topology. eL1 to eL3 represent major lineages. (**b**) Median-joining network. Circle sizes are proportional to haplotype frequency and connecting lines are proportional to mutation events between haplotypes (numbers of mutated positions are given for all except one mutation). Colours indicate geographical areas where the basidiomata were collected. (**c**) Statistical parsimony network. Parsimony probabilities were set at 95%. Sizes of circular and rectangular areas are proportional to the number of individuals with that haplotype. Ectomycorrhizal tree families co-occurring in the sampling sites are abbreviated as follows: B = Betulaceae, F = Fagaceae, P = Pinaceae, S = Salicaceae.

**Figure 4:**
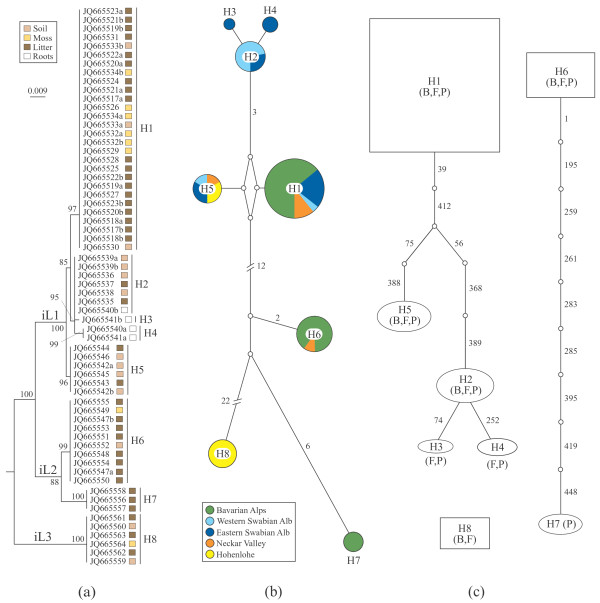
**Genetic variation based on 63 non-recombining ITS + 5.8S + D1/D2 haplotype sequences of *Sebacina incrustans.*** Haplotypes are coded with H1 to H8 and heterozygous sequences are coded with a and b. (**a**) Maximum likelihood phylogenetic tree. Tree topology was computed from 1000 runs and was midpoint rooted. Bootstrap supports (>50%) are shown for each node. Substrate types where the basidiomata grew are mapped on the topology. iL1 to iL3 represent major lineages. (**b**) Median-joining network. Circle sizes are proportional to haplotype frequency and connecting lines are proportional to mutation events between haplotypes (numbers of mutated positions are given for all except one mutation. Colours indicate geographical areas where the basidiomata were collected. (**c**) Statistical parsimony network. Parsimony probabilities were set at 95%. Sizes of circular and rectangular areas are proportional to the number of individuals with that haplotype. Ectomycorrhizal tree families co-occurring in sampling sites are abbreviated as follows: B = Betulaceae, F = Fagaceae, P = Pinaceae.

Genealogy analyses of ITS + 5.8S and/or D1/D2, as well as RPB2 and ATP6 did not support the morphological species *Sebacina epigaea* and *S. incrustans* as monophyletic taxa. Phylogenetic analyses of datasets with and without recombination blocks split both into identical major lineages. In *S. epigaea*, three highly divergent lineages (named eL1, eL2 and eL3) were inferred from the ITS + 5.8S + D1/D2 dataset (Figure 3; Additional files 1 and 2); the monophyly of eL1 was not supported in the RPB2 and ATP6 datasets, except for RPB2 inclusive recombinant blocks (data not shown). In *S. incrustans*, all loci strongly supported three highly divergent lineages, iL1, iL2 and iL3 (Figure 4; Additional files 3 and 4). Approximately 95% of the population samples represented the lineage eL1 and 60% the lineage iL1. The ILD and SH tests did not detect any significant incongruent phylogenetic signal within (with and without recombination blocks) or among genes.

### Genetic diversity and spatial distribution of lineages

**Figure 5:**
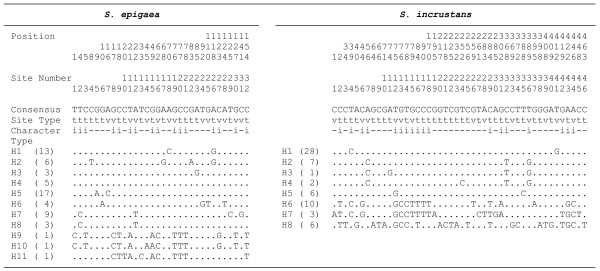
**Distribution of polymorphic sites after removing indels and recombination blocks in the ITS + 5.8S + D1/D2 haplotypes of *Sebacina epigaea *and *S. incrustans*.** Haplotype frequency is given in brackets. t = transitions, v = transversions, i = phylogenetically informative sites, – = uninformative sites.

Percentages of ITS + 5.8S + D1/D2 sequence divergence before and after removing recombination blocks were higher in *Sebacina epigaea* than in *S. incrustans* (Additional file 5). Most of the polymorphisms were localised at the third codon position for the RPB2 and ATP6 loci. The dN/dS ratios were below one, indicating that negative selection is the predominant force in RPB2 and ATP6 evolution (data not shown). For both species, a high proportion of population samples (approximately 30% in ITS + 5.8S + D1/D2 and 80% in RPB2) included one or more heterozygous positions. On the contrary, the ATP6 sequences lacked any heterozygous positions. Heterozygous positions ranged between 1 and 8 in *S. epigaea,* and between 1 and 11 in *S. incrustans* in the RPB2 datasets. With regard to the ITS + 5.8S + D1/D2 datasets, between 1 and 3 heterozygous positions were found in *S. epigaea,* and between 1 and 4 in *S. incrustans* (Additional file 6). The total number of recombination blocks for *S. epigaea* was 39 in ITS + 5.8S + D1/D2, 31 in RPB2 and 4 in ATP6, whereas for *S. incrustans,* 19 were detected in ITS + 5.8S + D1/D2, 31 in RPB2 and 3 in ATP6. After removing indels and recombination blocks, 11 ITS + 5.8S + D1/D2 haplotypes were found among the 50 population samples from *S. epigaea* and eight haplotypes in the 48 population samples for *S. incrustans*. Table 1 and Figure 5 summarise the statistics of nucleotide variation in the ITS + 5.8S + D1/D2 regions for both morphospecies, where the ITS region (ITS1 and ITS2) was identified as the most variable region. The neutrality tests performed had non-significant values in most cases; therefore, the equilibrium model of neutral evolution could be not rejected. Only for the whole sample of *S. incrustans* was significant value detected for Fu & Li’s D* test, suggesting background selection. Population samples from Bavarian Alps (BA) for both species yielded significant results for the Fu & Li’s D* and F* tests (Table 1).

**Table 1:** **Population statistics, diversity estimates and neutrality tests based on ITS + 5.8S + D1/D2 nucleotide sequences of *Sebacina epigaea *and *S. incrustans***

**Species**	**n**	**h**	**s**	**hd**	**k**	**π**	**θω**	**Tajima’s**	**Fu & Li’s**	
**Population**								**D**	D*	F*
***S. epigaea***										
BA	38	7	12	0.783	3.691	0.024	0.018	1.04	1.48*	1.57
WA	6	4	4	0.679	2.214	0.014	0.010	1.90	1.31	1.59
EA	2	1	0	0.000						
NV	3	2	13	0.500	6.500	0.042	0.046	−0.84	−0.84	−0.86
HO	1	1	1	1.000	1.000	0.006	0.006			
All	50	11	53	0.853	4.962	0.032	0.044	−0.87	1.07	0.42
***S. incrustans***										
BA	22	13	23	0.059	9.124	0.020	0.013	2.02	1.67*	2.09*
WA	6	3	7	0.667	3.238	0.007	0.001	0.69	0.60	0.67
EA	7	5	9	0.769	3.615	0.008	0.001	0.97	0.96	1.09
NV	5	3	18	0.700	7.600	0.016	0.019	−0.89	−0.89	−0.95
HO	8	2	25	0.429	10.714	0.023	0.021	0.59	1.61*	1.52
All	48	8	46	0.755	11.341	0.024	0.021	0.54	1.82*	1.60

Sampling areas are coded as follows: BA = Bavarian Alps, WA = Western Swabian Alb, EA = Eastern Swabian Alb, NV = Neckar Valley, HO = Hohenlohe. n = sample size, h = number of haplotypes, s = number of segregating sites, hd = haplotype diversity, k = average number of nucleotide pairwise differences, π = number of nucleotide differences per site, θω = Watterson’s estimate of ϕ per site. Significant tests (*P* < 0.05) are marked with *.

The distribution of population samples of *S*. *epigaea* indicates that eL1 has a wider distribution, while eL2 is restricted to Hohenlohe and eL3 to Neckar Valley (Figure 3). Within *S. incrustans*, iL1 has a wider geographic distribution, iL2 is restricted to Bavarian Alps and Neckar Valley, and iL3 to Hohenlohe (Figure 4).

### Demographic structure

Within each morphospecies, both network estimation approaches found essentially similar structure patterns between the ITS + 5.8S + D1/D2 (Figures 3 and 4), RPB2 (Additional files 1 and 3) and ATP6 (Additional files 2 and 4) haplotypes. The median-joining network revealed three frequent haplotypes (H1, H5, H7) in *Sebacina epigaea*, whereas haplotypes H9 and H10 were obtained from the same collection, and H11 represented a single population sample. All haplotypes were restricted to one or two sample areas (Figure 3). In the *S. incrustans* ITS + 5.8S + D1/D2 dataset, H1 was the most frequent haplotype, whereas H3 and H4 occurred at low frequency. H1 was scattered throughout Bavarian Alps, Eastern Swabian Alb, Western Swabian Alb, and Neckar Valley, and appeared to have an ancestral position to the other haplotypes (Figure 4). Statistical parsimony analysis for *S. epigaea* yielded three ITS + 5.8S + D1/D2 networks comprising eight connected (H1-H8: eL1), two connected haplotypes (H9, H10: eL2) and one single haplotype (H11: eL3). H5 had an ancestral position to H1-H4 and H6-H8 (Figure 3). RPB2 and ATP6 genes produced similar network structures (Additional files 1 and 2). Analysis of the ITS + 5.8S + D1/D2 dataset for *S. incrustans* resulted in one network with five haplotypes (H1-H5: iL1), one with two haplotypes (H6, H7: iL2) and one with a single haplotype (H8: iL3). RPB2 and ATP6 genes produced nearly identical network structures (Additional files 3 and 4). In general, analyses of RPB2 from both morphospecies showed a greater number of haplotypes in comparison with ITS + 5.8S + D1/D2 dataset. Conversely, a smaller number of haplotypes was detected in ATP6 dataset. When we performed nested clade analysis using ITS + 5.8S + D1/D2 datasets containing recombination blocks within *S. epigaea,* a total of nine unconnected networks were detected, whereas S*. incrustans* yielded six unconnected networks (see Additional file 5).

In *S. epigaea*, only eL1 was significantly separated from eL2 and eL3, whereas in *S. incrustans*, all pairs of lineages had significant pairwise F_ST_ values and exact tests (Table 2a). Most of the pairwise F_ST_ values and exact tests between pairs of sampling areas were significant in *S. epigaea* and a minor number of pairs of sampling areas proved to be significant for *S. incrustans* (Table 2b). Mantel tests comparing genetic differentiation (F_ST_) and geographical distance matrices were not significant (data not shown). AMOVA indicated non-significant differentiation among groups for both scenarios analysed, whereas percentages of total variance were significant among populations within groups and within populations (Table 3).

**Table 2:** **Results of exact tests (above the diagonal line) and pairwise F**_**ST **_**values and their significance (below the diagonal line) based on ITS + 5.8S + D1/D2 sequences of *Sebacina epigaea *and *S. incrustans***

			***S. epigaea***					***S. incrustans***			
(a)	eL1	eL2	eL3				iL1	iL2	iL3		
eL1		***	*			iL1		***	***		
eL2	0.78***					iL2	0.80***		***		
eL3	0.71*	0.85				iL3	0.85***	0.91***			
(b)	BA	WA	EA	NV	HO		BA	WA	EA	NV	HO
BA		***	***		**	BA		***	***		**
WA	0.14*		**	*		WA	0.81***				
EA	0.55***	0.74***		*		EA	0.50***	0.07		*	
NV	0.21*	0.36**	0.61*			NV	0.36	0.41*	0.11		
HO	0.78***	0.87*	0.99	0.63		HO	0.98***	0.51	0.32	0.53	

(a) Between pairs of lineages (L), (b) between pairs of collection areas, that are coded as follows: BA = Bavarian Alps, WA = Western Swabian Alb, EA = Eastern Swabian Alb, NV = Neckar Valley, HO = Hohenlohe. Significant exact tests and F_ST_ values are indicated with asterisks: * *P* < 0.05, ** *P* < 0.01, *** *P* < 0.001.

**Table 3:** **Summary of analyses of molecular variance (AMOVA) based on ITS + 5.8S + D1/D2 haplotype sequences of *Sebacina epigaea *and *S. incrustans***

	***S. epigaea***			***S. incrustans***		
**Scenario**	AG	WG	WP	AG	WG	WP
(i) Geographic distribution	−2.07	**51.41**	**51.29**	37.93	**3.48**	**58.59**
North [HO] vs. center [NV,WA,EA] vs. south [BA]						
(ii) Geographic barrier	−51.38	**97.01**	**53.73**	−11.67	**47.80**	**63.87**
North [NV,WA,EA,HO] vs. south of the Swabian Alb [BA]						

Two grouping scenarios were tested (see Methods). Sampling areas are coded as follows: BA = Bavarian Alps, WA = Western Swabian Alb, EA = Eastern Swabian Alb, NV = Neckar Valley, HO = Hohenlohe. AG = Percentage of total variance among groups, WG = Percentage of total variance among populations within groups, WP = Percentage of total variance within populations. Bolded values are significant (*P* < 0.001)..

### Morphological and ecological traits

**Figure 6:**
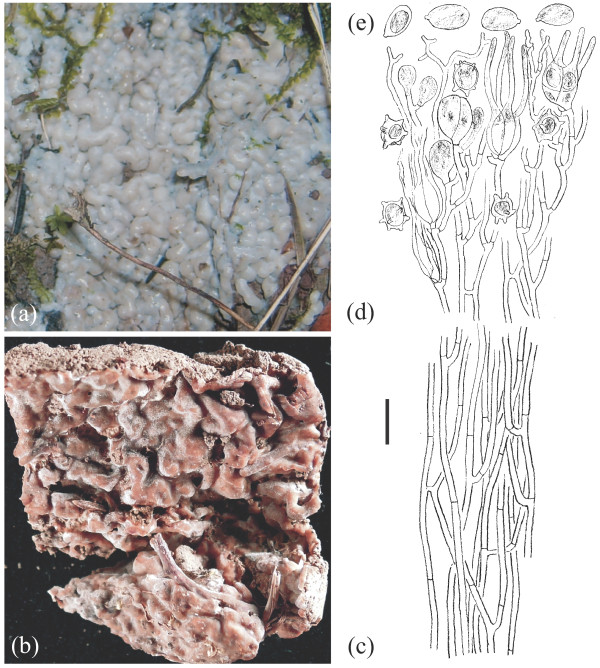
**Basidioma morphology of *Sebacina epigaea *eL1 (TUB 019821).** (**a**) Fresh specimen. (**b**) Dried specimen. Longitudinal section through a basidioma. (**c**) Trama. (**d**) Hymenium with resting spores. (**e**) Basidiospores. Bar = 10 μm.

**Figure 7:**
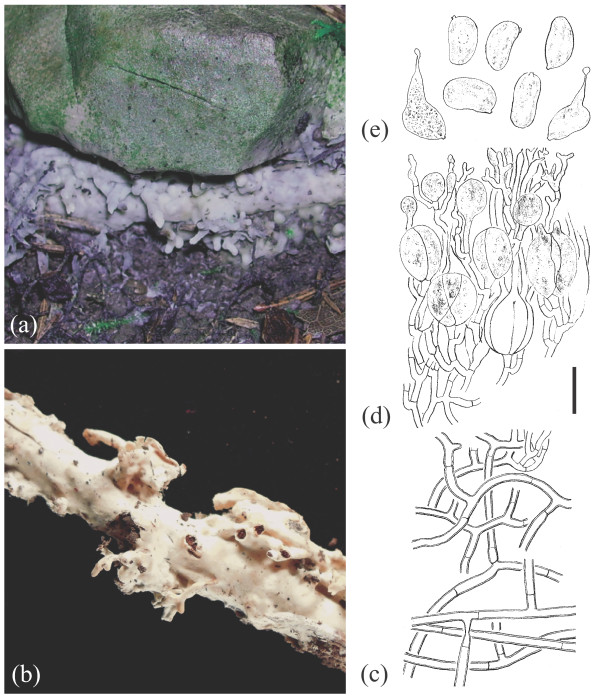
**Basidioma morphology of *Sebacina incrustans *iL1 (TUB 019608).** (**a**) Fresh specimen. (**b**) Dried specimen. Longitudinal section through a basidioma. (**c**) Trama. (**d**) Hymenium. (**e**) Basidiospores. Bar = 10 μm.

**Figure 8:**
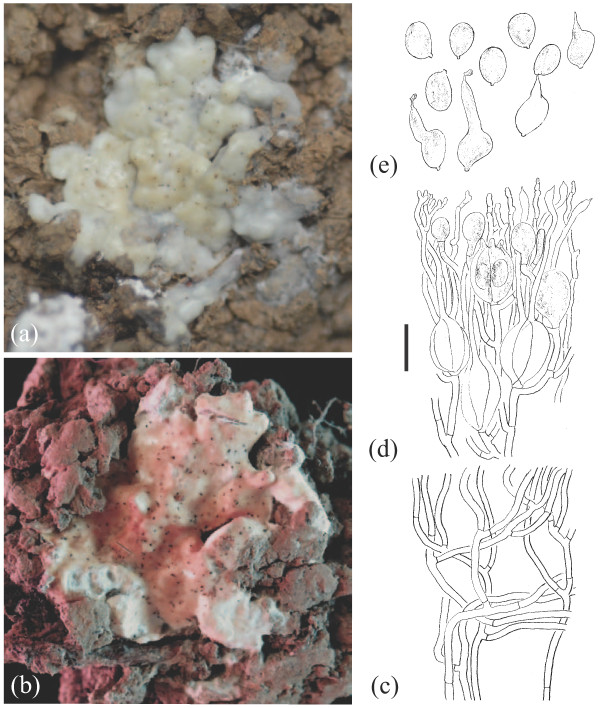
**Basidioma morphology of *Sebacina incrustans *iL2 (TUB 019641).** (**a**) Fresh specimen. (**b**) Dried specimen. Longitudinal section through a basidioma. (**c**) Trama. (**d**) Hymenium. (**e**) Basidiospores. Bar = 10 μm.

**Figure 9:**
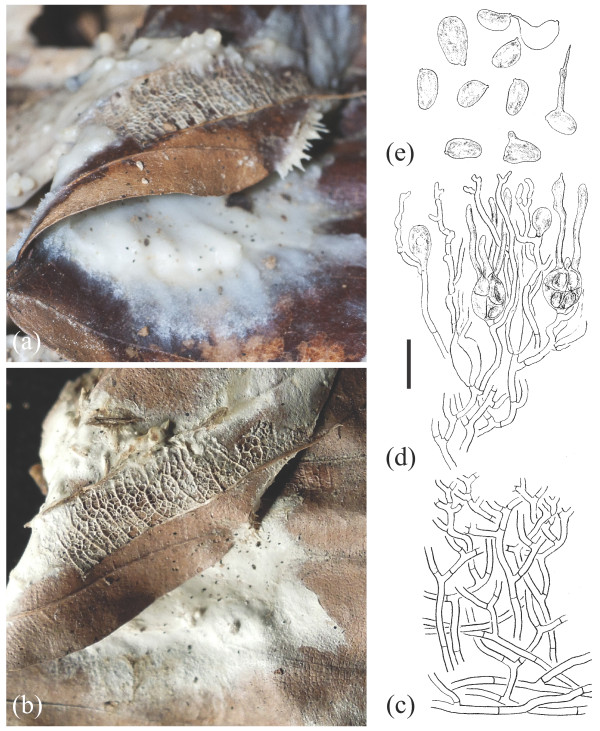
**Basidioma morphology of *Sebacina incrustans *iL3 (TUB 019648).** (**a**) Fresh specimen. (**b**) Dried specimen. Longitudinal section through a basidioma. (**c**) Trama. (**d**) Hymenium. (**e**) Basidiospores. Bar = 10 μm.

Main morphological and ecological traits analysed from both morphospecies are summarized as follows (see Figures 6,7,8,9 and Additional file 6):

i. **Habitat and habit of basidiomata.** Population samples within *Sebacina epigaea* showed low phenotypic variation, whereas populations in *S. incrustans* displayed high levels of basidiomata plasticity that often were linked with the substrate on which they were growing and their age. Basidiomata of *S. epigaea* growing on naked soil with vertical exposure and on litter produced thin to 2 mm thick basidiomata of gelatinous consistency, which are smooth or meruloid and which often became translucent. Basidiomata of *S. incrustans* growing on diverse plant substrates are resupinate, up to 3 mm thick, smooth and cartilaginous, whereas those on naked soil have a habit reminiscent to *S. epigaea*.

ii. **Colour of fresh and dried basidiomata.** Fresh basidiomata of *S. epigaea* are uniformly gray and opalescent. Old and dried basidiomata in *S. epigaea* become membranous and brownish or greyish, and were often hard to see on the substrate. Both fresh and dried basidiomata in *S. incrustans* that grow on herbaceous substrates are typically whitish to cream or ochraceous; but the basidiomata that grow directly on naked soil have a colour similar to *S. epigaea,* but become yellow after drying.

iii. **Microscopic characteristics.** There was no variation in microscopic characteristics among populations within *S. epigaea* eL1 (eL2 and eL3 containing only each one sample were not microscopically analysed). In *S. incrustans*, however, there was some differentiation of certain microscopic features, for example, wall thickness of the trama hyphae and the size of the basidia and basidiospores.

iv. **Ecology.** Basidiomata of both morphological species were mainly collected from forests with trees in the families Fagaceae (*Fagus sylvatica*, *Quercus robur*) and Pinaceae (*Abies alba*, *Larix decidua*, *Picea abies, Pinus sylvestris*), but some in vegetation with Betulaceae (*Carpinus betulus*, *Corylus avellana*) and Salicaceae (*Salix appendiculata*). Within *S. epigaea,* eL1 and eL3 seem to be rather restricted to *Picea abies* and eL2 occurred in a forest composed of frondose trees (*Carpinus betulus*, *Corylus avellana, Fagus sylvatica, Quercus robur*).

Additional aspects of morphology, ecology and phylogenetic diversity within *S. epigaea* and *S. incrustans* are described and discussed in Additional file 7.

## Discussion

### Intraspecific genetic variability at the regional scale

Although our sampling area was rather geographically restricted, we found high levels of genetic diversity within *Sebacina epigaea* and *S. incrustans* morphospecies, in contrast with the findings of Nilsson *et al.*[16]. There were two major factors that can explain some of the genetic diversity: (i) recombination events and (ii) high frequency of synonymous mutations. The first factor was detected previously by Vandenkoornhuyse *et al.*[17] for arbuscular mycorrhizal fungi, and could explain the high sequence diversity found in the ITS regions of environmental samples in Sebacinales, see e.g. [11]. The high number of recombination events detected in this study, particularly between populations in *S. epigaea*, suggests that sexuality has an influence on the patterns of genetic divesity and speciation at a regional geographic scale. However, because both morphological species are obligate mycorrhizal formers and cannot be grown in axenic culture, mating experiments are not possible, and therefore we cannot determine whether or not they represent the same or different biological species. The second factor of DNA variation comprised ~95% of the observed DNA polymorphisms in RPB2 and ~60% in the ATP6 sequences, and was driven by negative selection in which rare, deleterious alleles are removed from a population. The evolutionary significance of this type of selection is still not well understood, but there is an indication that some synonymous polymorphisms are involved in functional roles [18]. We hypothesise that intraspecific genetic variation provide Sebacinales symbionts the opportunity to select genotypes that adapt more rapidly, e.g. those that are physiologically adapted to environmental heterogeneity and/or host availability [19]. The total genetic variation of our data, however, cannot be completely explained by the evolutionary processes discussed above.

Gene genealogies revealed three genetic divergent lineages within each morphospecies; interestingly, some of them were detected within the same site separated by only a few centimeters. We suggest that these patterns of geographical distribution are driven by environmental discontinuities and/or heterogeneity in resources, as suggested by Gryta *et al.*[20]. However, further study is necessary to determine if highly divergent co-occurring lineages within *Sebacina epigaea* and *S. incrustans* have evolved in sympatry or whether current patterns of geographic distribution are rather the result of recent dispersal events.

The ITS + 5.8S + D1/D2 regions contain significant polymorphisms that may resolve lower and higher relationships across the Sebacinales, even after removing recombinant segments. In particular, the ITS region was useful for discriminating among populations and we therefore propose it as a potentially useful DNA segment for barcoding in the Sebacinales.

### Concordance of nuclear and mitochondrial genealogies

Unlike other fungus taxa that have been affected by natural recombination (see, for example, [5,21,22]), topologies of the genealogies inferred from two nuclear regions (ITS + 5.8S + D1/D2 and RPB2) and one mitochondrial region (ATP6) clearly identified three distinctive lineages in both the *Sebacina epigaea* and *S. incrustans* morphospecies, and these were significantly concordant. The concordance between nucDNA and mtDNA sequence data may indicate either complete lineage sorting or an ancient diversification [23,24].

### Circumscribing major lineages

Surprisingly, both morphological species displayed relatively similar lineage/haplotype structures: a frequent and more widely distributed lineage and two small lineages. *Sebacina epigaea* eL1 included population samples from Estonia and *S. incrustans* iL1 included population samples from Austria and Denmark. Sampling bias is a possible explanation for the presence of small, distinct lineages (eL2, iL2, eL3 and iL3). Lineages/cryptic species within *S. epigaea* seems to have involved large evolutionary divergence accompanied by only a few changes in morphology and microscopic characteristics. The lack of morphological innovations among the populations analysed could suggest that diversification may be driven by ecological opportunities offered by the availability of new habitats and/or host plants. Host specialisation and host switches have been considered to be important drivers for speciation [21]. However, in this study, no obvious indication of substrate and host specialisation could be detected in circumscribing the major lineages (Figure 2; Additional file 6). Because most of the collection sites had more than one potential host tree for both morphospecies, further study focusing on the molecular analysis of ectomycorrhizal root tips should allow us to find a link between mycobionts and host tree(s). In *S. incrustans*, populations exhibited enormous plasticity in basidioma habit, which was linked with the type of substrate upon which they were formed. Such variation was observed even within the same haplotype.

Nuclear and mitochondrial data showed evidence of gene flow between populations within major lineages in *S. epigaea* and *S. incrustans* morphospecies. We did not find evidence of an isolation-by-distance model for population structure. The Swabian Alp does not seem to represent a geographical barrier for wind or animal dispersal. In addition, anthropogenic dispersal of spores and/or mycelia contained in the soil and roots should not be underestimated as means of dispersal. An important putative scenario could be that these mycobionts have acquired their current geographical distribution through extensive planting of *Picea abies* across Europe.

The type specimens of *Sebacina epigaea* and *S. incrustans* are more than 150 years old and catalogued as historical collections; therefore it is not possible to study the material or extract DNA from the basidiomata. Genealogies inferred from nucDNA and mtDNA genes strongly support divergent lineages within these morphospecies. However, considering the low morphological differentiation within and between lineages, we suggest that sampling specifically from the type localities in France and Great Britain of these species would increase the probability of more accurately defining each lineage in respect to its type specimen.

## Conclusions

Our results indicate that the high intraspecific genetic diversity within the morphospecies *Sebacina epigaea* and *S. incrustans* is mainly the outcome of natural recombination events and synonymous mutations. Phylogenetic and demographic inferences from nuclear and mitochondrial loci support the division of each morphological species into three distinct phylogenetic lineages, which seem to have evolved independently for a long time. None of these putative cryptic species appears to be linked to geographical provenance, host preference or morphological innovations. However, future studies correlating genetic diversity across lineages with differences in ecological niches may help us to better understand the factors shaping speciation in these morphological species.

## Methods

### Population sampling

Basidiomata of *Sebacina epigaea* (Berk. & Broome) Bourdot & Galzin and *S. incrustans* (Pers.) Tul. were collected in Southern Germany from August to October 2010 and 2011 from plots in diverse forest stands that were between 20 km and 250 km apart from each other (Figure 1). Collection areas are categorised as follows: (1) the Bavarian Alps (BA) included five sites separated from each other by a few hundred meters to 20 km. Site BA1 is a pure montane *Picea abies* plantation with a northern exposure. BA2, BA3 and BA4 sites are dominated by montane mixed forests composed mainly of *P. abies*. Site BA5 is a *Fagus*-dominated mixed forest on a lakeside, separated by 20 km from the remaining BA sites. (2) The Western Swabian Alb (WA) included three sites, each separated by 1 km, comprising a submontane slope with mixed forests dominated by *Fagus sylvatica* with a south-western exposure (WA1) and an eastern exposure (WA3). Site WA2 is a plateau dominated by old specimens of *Abies alba*. (3) The Eastern Swabian Alb (EA) included two sites (EA1 and EA2) in a planar landscape separated by 1 km. Both sites contain mixed forests dominated by old trees of *Fagus sylvatica*. (4) The Neckar Valley (NV) included six sites separated by a few hundred meters to 30–10 km. Sites NV1, NV2 and NV3 comprise mixed forest separated from each other by 200 m (NV1 is located on a small riparian lake). Sites NV4 and NV5 correspond to a plantation of *Picea abies,* and the sampling plots were separated by 2 km. Site NV6 represents a coniferous forest dominated by *Pinus sylvestris.* (5) Hohenlohe (HO) included two sites separated by 50 m within a mixed forest dominated by *Carpinus betulus* in a moist basin.

For each collection site, details such as the location, altitude, substrate and ectomycorrhizal plant species nearby were recorded (Additional file 6).

### DNA extraction, PCR amplification and sequencing

Total genomic DNA was extracted from dried basidioma fragments using the InnuPREP Plant DNA Kit (Analytik Jena, Jena, Germany) following the standard protocol. Fungal portions contained in Eppendorf tubes were deep-frozen in liquid nitrogen and then ground several times with a sterile plastic pestle.

The internal transcribed spacer (ITS1 and ITS2), 5.8S and D1/D2 regions of the nuclear ribosomal DNA were amplified with the primer combination ITS1F and NL4 (for oligonucleotide primer sequences, see Additional file 8). PCRs were performed in a total volume of 25 μl, containing 5.00 μl GC buffer 5x (including 7.5 mM MgCl_2_), 15.25 μl water, 1.00 μl dNTP mix (5 mM), 0.50 μl of each primer (25 pmol/μl), 0.25 μl Phusion™ High-Fidelity DNA Polymerase (Finnzymes Oy, Vantaa, Finland) (2 U/μl) and 2.50 μl undiluted DNA. PCR amplification was conducted as follows: 35 cycles of 10 s at 98°C, 20 s at 55°C and 30 s at 72°C, with a final extension of 10 min at 72°C. In the case of negative or weak amplification, PCRs were repeated as follows: 5.00 μl buffer 10x, 0.75 mM MgCl_2_ (50 mM), 14.50 μl water, 1.00 μl dNTP mix (5 mM), 0.50 μl of each primer (25 pmol/μl), 0.25 μl Mango*Taq*™ DNA Polymerase (Bioline, Luckenwalde, Germany) (2 U/μl), and undiluted 2.50 μl DNA under the following cycling profiles: 10 cycles of 30 s at 94°C, 45 s at 60°C (−1°C per cycle), and 75 s at 72°C, followed by 26 cycles of 30 s at 94°C, 45 s at 50°C and 75 s at 72°C, with a final extension of 7 min at 72°C.

The second largest subunit of RNA polymerase II (RPB2) regions 5–7 was amplified with the primer combination fRPB2-5F and bRPB2-7.1R using Phusion DNA Polymerase and the PCR conditions indicated above. Subsequently, samples that yielded no or only weak products were amplified with Mango*Taq* Polymerase and the primer pairs bRPB2-5F and bRPB2-7.1R, or sRPB2-5.1F and sRPB2-7R following the thermocycling program by Matheny [25].

The mitochondrial adenosine triphosphatase subunit 6 (ATP6) was amplified using the primer combinations ATP6-3/ATP6-4 or sATP6-3/ATP6-4 and Mango*Taq* Polymerase with the PCR concentration reactions indicated above and the cycling profiles described by Kretzer and Bruns [26].

The presence of PCR products was monitored by 0.7% agarose gel at 140 V for 15 min in 1× Tris-acetate-EDTA buffer and made visible by ethidium bromide staining and UV light at 254 nm wavelength. The amplified DNA fragments were purified using a ExoSAP-IT® reagent (USB Corporation, Cleveland, OH, USA) diluted 1:20 according to the manufacturer’s instructions. Purified PCR products were cycle sequenced in both directions with a 1:6 BigDye Terminator v3.1 Cycle Sequencing Kit (Applied Biosystems, Foster City, CA, USA) on an ABI Prism 3130*xl* Genetic Analyzer (Applied Biosystems). The primers used for DNA sequencing in both directions are listed in Additional file 8. Fungal vouchers/basidiomata used in this study are deposited in the Herbarium Tubingense (TUB; Additional file 6).

### Sequence editing, identity and alignments

Forward and reverse sequence fragments were assembled and edited using Sequencher 4.10.1 (Gene Codes Corporation, Ann Arbor, MI, USA). The ITS + 5.8S + D1/D2 and RPB2 sequences were screened against those available in the GenBank database (http://www.ncbi.nlm.nih.gov) using the Basic Local Alignment Search Tool (BLAST; [27]). ATP6 sequences were analysed with Basidiomycota sequences from our lab (unpublished data by SG) because there are no available sequences in GenBank for Sebacinales.

In our study, phylogenetic and demographic population analyses were inferred from morphological species concepts. We assembled seven datasets: datasets 1 and 2, containing 63 *Sebacina epigaea* and 63 *S. incrustans* ITS + 5.8S + D1/D2 sequences which were automatically aligned using MAFFT 6.815b under the E-INS-i algorithm [28] and POA 2 [29]; datasets 3 and 4, including 85 (*S. epigaea*) and 78 (*S. incrustans*) RPB2 (exon 6) sequences aligned with MAFFT; datasets 5 and 6, comprising 11 (*S. epigaea*) and 42 (*S. incrustans*) ATP6 sequences aligned using MAFFT; and dataset 7, containing our own ITS haplotype sequences of both species, and those from GenBank and UNITE [30]. Datasets containing sequences obtained from *Sebacina* basidiomata and ectomycorrhizae from Europe were aligned using MAFFT und POA. For datasets 1, 2 and 7, selection of the most consistent alignments (MAFFT) was performed using trimAl 1.4 [31]. Nucleotide alignments from datasets 3 to 6 were improved manually from amino acid codon sequences in Se-Al 2.0a11 Carbon [32].

The DNA sequences (original datasets including heterozygous sequences) used in this study have been submitted to GenBank (accession numbers JQ665465–JQ665564 for ITS, 5.8S and D1/D2; JQ665565–JQ665657 for RPB2 exon 6; and JQ665658–JQ665710 for ATP6). Alignments are deposited at TreeBASE under submission ID S13159.

### Haplotype reconstruction, detecting recombination and selection pressure

Because all sequences are derived from dikaryotic (n + n) isolates, we used PHASE 2.1 [33,34] as implemented in DnaSP 5.10.01 [35] using the Markov Chain Monte Carlo option followed by 1000 iterations under a hybrid model to infer haplotype phases. From haplotype sequences within each species, we removed indels and excluded infinite-site violations using Map as implemented in SNAP Workbench 2.0 [36,37]. Population recombination parameters were estimated following the method of Hudson and Kaplan [38] based on the minimum number of recombination events in a sample (*R*_M_) using DnaSP and RecMin [39] as implemented in SNAP Workbench. The significance of the R_M_ estimation was calculated by performing 10,000 coalescent simulations [40] in DnaSP. Estimations of the selection pressure on coding sequences were based on the ω = dN/dS ratio by comparing the rates of non-synonymous and synonymous mutations. We calculated ω ratios using the Synonymous Non-synonymous Analysis Program (SNAP) [41].

### Phylogenetic relationships, congruence among gene phylogenies, neutrality tests and demographic structure analyses

Maximum likelihood analysis with combined rapid bootstrapping under the GTRCAT model was computed from 1000 runs with RAxML 7.0.4 [42,43]. The phylogenetic trees were midpoint rooted using FigTree 1.3.1[44].

Conflicting phylogenetic signals of the different datasets were checked using a partition homogeneity test/incongruence length difference (ILD) test [45] as implemented in PAUP* [46]. The number of ILD replicates was set to 1000, setting one tree per replicate and branch swapping with tree bisection–reconnection (TBR). In order to test if the topologies of the different analyses and datasets were significantly different we used the maximum parsimony based Shimodaira-Hasegawa (SH) test as implemented in PAUP, using 1000 replicates with TBR swapping.

DnaSP was used to calculate the standard indices of population diversity for each lineage and sampling area. To detect departures from a constant population size under the neutral model, Tajima’s D [47], and Fu’s and Li’s D* and F* [48] statistics were calculated using Arlequin 3.5.1.2 [49]. The significance of these values was obtained in neutrality tests with 1000 permutations.

Intraspecific gene genealogies were inferred using the median joining [50] and statistical parsimony networks. Haplotype genealogies were constructed using Network 4.6.1 (http://www.fluxus-engineering.com) with the parameter ϵ = 10 and the ‘MP’ option to identify and eliminate unnecessary median vectors and links [51]. Network graphics were generated using Network Publisher 1.3 (http://www.fluxus-engineering.com). Analyses of genetic differentiation among species using a 95% parsimony limit reconstruction criterion [52] suggest that biological species often form unconnected parsimony networks. Based on these measurements, we reconstructed a parsimony network of haplotypes with a 95% connection probability limit using TCS 1.21 [53]. These analyses were run separately for sequences with and without recombination blocks. To examine the amount of genetic structure within and among population groupings, hierarchical analyses of molecular variance (AMOVA; [54-56]) were conducted in Arlequin. For this purpose, we tested two hypothetical scenarios: (i) differentiation explained by geographic distribution: North (HO) vs. Central (WA, EA, NV) vs. South (BA) (scenario 1); and (ii) differentiation between populations separated by a geographic barrier. For this, we compared populations from the north of the Swabian Alb (WA, EA, NV, HO) vs. those south of the Swabian Alb (BA) (scenario 2). To search for genetic differentiation between population samples and major lineages, exact tests of population differentiation [57] and F_ST_ values were computed in Arlequin. The significance of these estimators was assessed using 1000 permutations. Furthermore, we examined whether a correlation existed between genetic (F_ST_) and geographic distance matrices with a Mantel test using zt 1.1 [58].

### Light microscopy

The microscopic structures of the basidiomata were analysed with a light microscope using standard procedures. Microscopic structures were analysed from dried specimens using 3% KOH and stained with aqueous 1% phloxine. Measurements of basidiospores (*n* = 51) are given as length and width, uncommon extreme values appear in brackets. Basidiospore length/width quotient (Q), mean and standard deviation were calculated.

## Competing interests

The authors declare that they have no competing interests.

## Authors’ contributions

KR and SG conceived and designed the study and wrote the manuscript, KR generated the sequences; FO performed specimen drawings and SG made microscopic descriptions; KR, RB, FO and SG revised several versions of this manuscript. All authors read and approved the final manuscript.

## Supplementary Material

Additional file 1 **Genetic variation based on 85 non-recombining RPB2 sequences of *Sebacina epigaea.*** Haplotypes (H) are defined from ITS + 5.8S + D1/D2 dataset, additional haplotypes found in the RPB2 are encoded with letters and heterozygous sequences are coded with a and b. (**a**) Maximum likelihood phylogenetic tree. The tree topology was computed from 1000 runs and was midpoint rooted. Bootstrap supports (>50%) are shown for each node. Substrate types for the basidiomata are mapped on the topology. eL1 to eL3 represent major lineages. (**b**) Median-joining network. Circle sizes are proportional to haplotype frequency and connecting lines are proportional to mutation events between haplotypes (numbers of mutated positions are given for all except one mutation). Colours indicate geographical areas where the basidiomata were collected. (**c**) Statistical parsimony network. Parsimony probabilities were set at 95%. Sizes of circular and rectangular areas are proportional to the number of individuals with that haplotype. Ectomycorrhizal tree families co-occurring in sampling sites are abbreviated as follows: B = Betulaceae, F = Fagaceae, P = Pinaceae, S = Salicaceae. Click here for file

Additional file 2 **Genetic variation based on 9 non-recombining ATP6 sequences of *Sebacina epigaea.*** Haplotypes (H) are defined from ITS + 5.8S + D1/D2 dataset. (**a**) Maximum likelihood phylogenetic tree. The tree topology was computed from 1000 runs and midpoint rooted. Bootstrap supports (>50%) are shown for each node. Substrate types for the basidiomata are mapped on the topology. eL1 and eL3 represent main lineages. (**b**) Median-joining network. Circle sizes are proportional to haplotype frequency and connecting lines are proportional to mutation events between haplotypes (numbers of mutated positions are given for all except one mutation). Colours indicate geographical areas where the basidiomata were collected. (**c**) Statistical parsimony network. Parsimony probabilities were set at 95%. Sizes of circular and rectangular areas are proportional to the number of individuals with that haplotype. Ectomycorrhizal tree families co-occurring in sampling sites are abbreviated as follows: B = Betulaceae, F = Fagaceae, P = Pinaceae. Click here for file

Additional file 3 **Genetic variation based on 78 non-recombining RPB2 sequences of *Sebacina incrustans.*** Haplotypes (H) are defined from ITS + 5.8S + D1/D2 dataset, additional haplotypes found in the RPB2 are encoded with letters and heterozygous sequences are coded with a and b. (**a**) Maximum likelihood phylogenetic tree. The tree topology was computed from 1000 runs and was midpoint rooted. Bootstrap supports (>50%) are shown for each node. Substrate types for the basidiomata are mapped on the topology. iL1 to iL3 represent main lineages. (**b**) Median-joining network. Circle sizes are proportional to haplotype frequency and connecting lines are proportional to mutation events between haplotypes (numbers of mutated positions are given except for all one mutation). Colours indicate geographical areas where the basidiomata were collected. (**c**) Statistical parsimony network. Parsimony probabilities were set at 95%. Sizes of circular and rectangular areas are proportional to the number of individuals with that haplotype. Distributions of ectomycorrhizal tree families co-occurring in sampling sites are abbreviated as follows: B = Betulaceae, F = Fagaceae, P = Pinaceae. Click here for file

Additional file 4 **Genetic variation based on 42 non-recombining ATP6 sequences of *Sebacina incrustans.*** (**a**) Haplotypes (H) are defined from ITS + 5.8S + D1/D2 dataset and additional haplotypes found in ATP6 are encoded with apostrophes. Maximum likelihood phylogenetic tree. The tree topology was computed from 1000 runs and midpoint rooted. Bootstrap supports (>50%) are shown for each node. Substrate types for the basidiomata are mapped on the topology. iL1 to iL3 represent main lineages. (**b**) Median-joining network. Circle sizes are proportional to haplotype frequency and connecting lines are proportional to mutation events between haplotypes (numbers of mutated positions are given for all except one mutation). Colours indicate geographical areas where the basidiomata were collected. (**c**) Statistical parsimony network. Parsimony probabilities were set at 95%. Sizes of circular and rectangular areas are proportional to the number of individuals with that haplotype. Ectomycorrhizal tree families co-occurring in sampling sites are abbreviated as follows: B = Betulaceae, F = Fagaceae, P = Pinaceae. Click here for file

Additional file 5 **Genetic variation of ITS + 5.8S + D1/D2 haplotype sequences inferred from complete (Rec +) and after (Rec -) removing recombination blocks for *Sebacina epigaea* and *S. incrustans* datasets.** Genetic divergence represents uncorrected *p*-distances using Mesquite 2.75 [59]. Numbers of unconnected networks based on parsimony networks with a 95% connection probability limit using TCS [53]. n+n = number of dikaryotic samples, n = number of haplotype sequences. Click here for file

Additional file 6 **Population samples used in this study.** Respective Herbarium Tubingense (TUB) numbers, GenBank accession numbers, numbers of heterozygous sites, collection sites, altitude (meters above sea level), substrate type, putative ectomycorrhizal (ECM) tree(s), haplotype designations and assignment to main lineages are given. Note: haplotypes are defined from ITS datasets; additional haplotypes found in the RPB2 and ATP6 datasets are encoded with letters and apostrophes, respectively. Abbreviations of host trees: AB = *Abies alba*, CA = *Carpinus betulus*, CO = *Corylus avellana*, FA = *Fagus sylvatica*, LA = *Larix decidua*, PC = *Picea abies*, PN = *Pinus sylvestris*, QU = *Quercus robur*, SA = *Salix appendiculata*. Click here for file

Additional file 7 **Descriptive part of major phylogenetic lineages detected within *Sebacina epigaea* and *S. incrustans.*** Click here for file

Additional file 8 **List of primers and their nucleotide sequence used in this study.** For primer references see [60-64]. Primers marked with asterisk are only used for DNA sequencing. Click here for file
