# Supplementary figures and images for: High genetic diversity at the regional scale and possible speciation in Sebacina epigaea and S. incrustans
Source: BMC Evol Biol. 2013 May 22;13:102. doi: 10.1186/1471-2148-13-102 (PMC3665632; doi:10.1186/1471-2148-13-102)

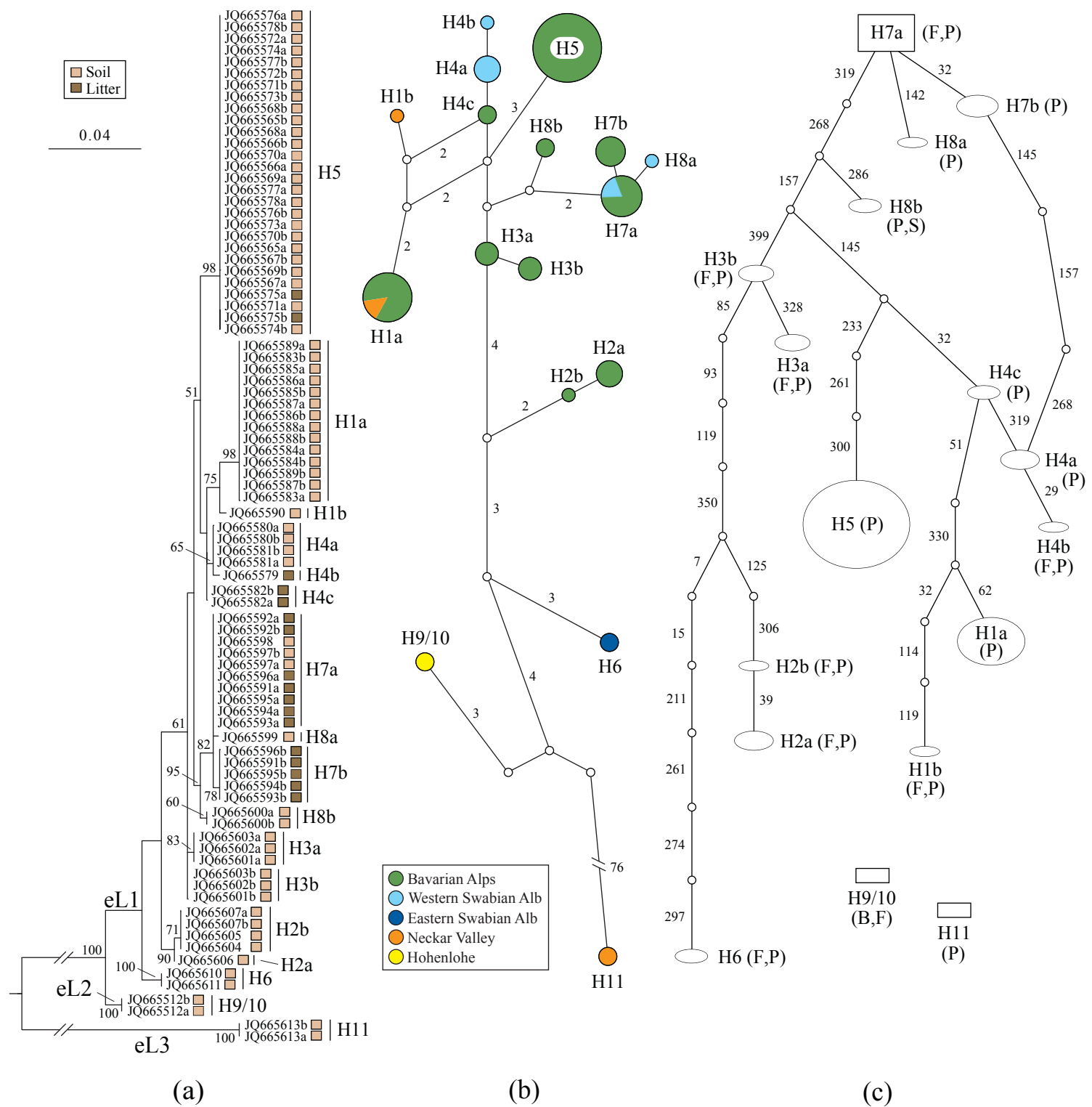

Supplement: Additional file 1 — Genetic variation based on 85 non-recombining RPB2 sequences of Sebacina epigaea. Haplotypes (H) are defined from ITS + 5.8S + D1/D2 dataset, additional haplotypes found in the RPB2 are encoded with letters and heterozygous sequences are coded with a and b. (a) Maximum likelihood phylogenetic tree. The tree topology was computed from 1000 runs and was midpoint rooted. Bootstrap supports (>50%) are shown for each node. Substrate types for the basidiomata are mapped on the topology. eL1 to eL3 represent major lineages. (b) Median-joining network. Circle sizes are proportional to haplotype frequency and connecting lines are proportional to mutation events between haplotypes (numbers of mutated positions are given for all except one mutation). Colours indicate geographical areas where the basidiomata were collected. (c) Statistical parsimony network. Parsimony probabilities were set at 95%. Sizes of circular and rectangular areas are proportional to the number of individuals with that haplotype. Ectomycorrhizal tree families co-occurring in sampling sites are abbreviated as follows: B = Betulaceae, F = Fagaceae, P = Pinaceae, S = Salicaceae. [file 1471-2148-13-102-S1.pdf]

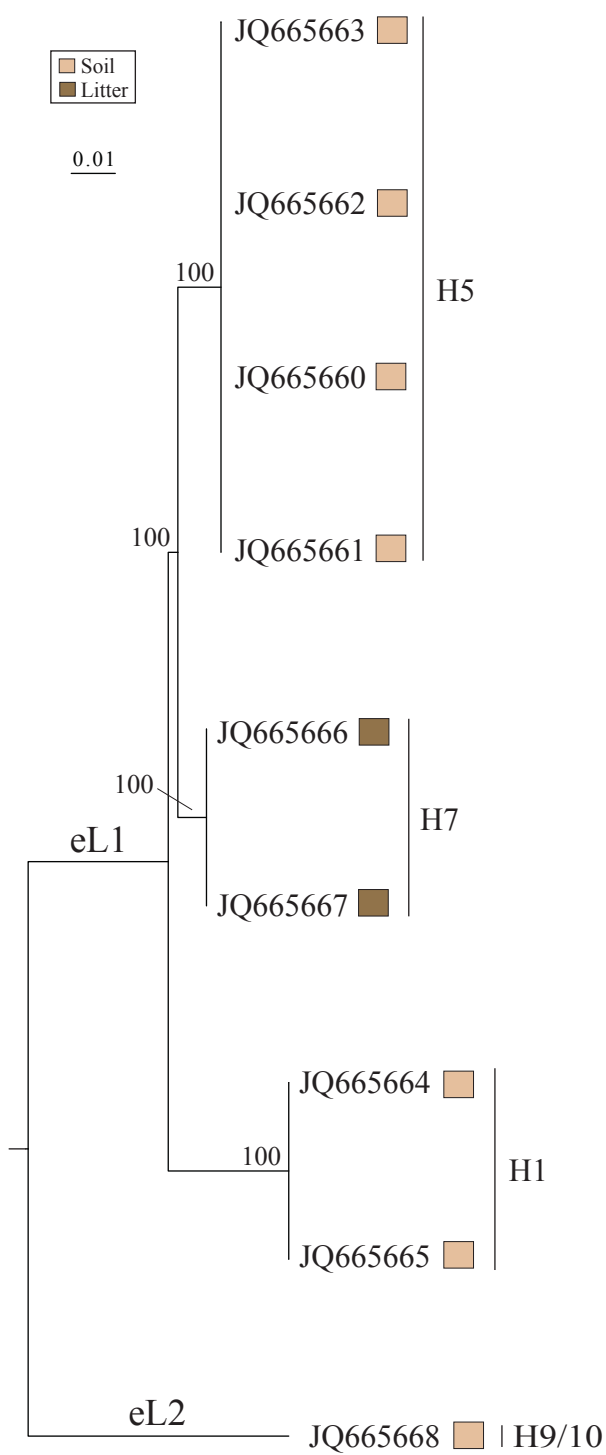

(a)

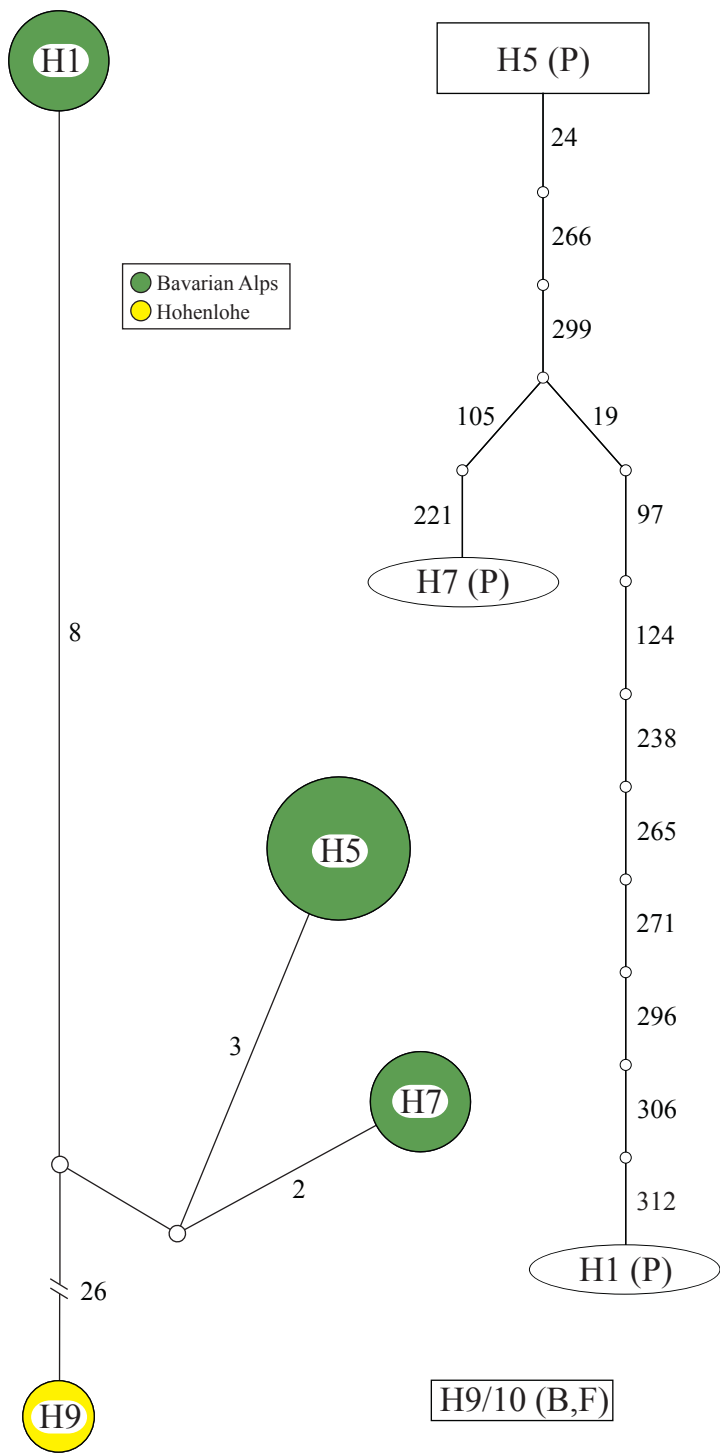

(b)

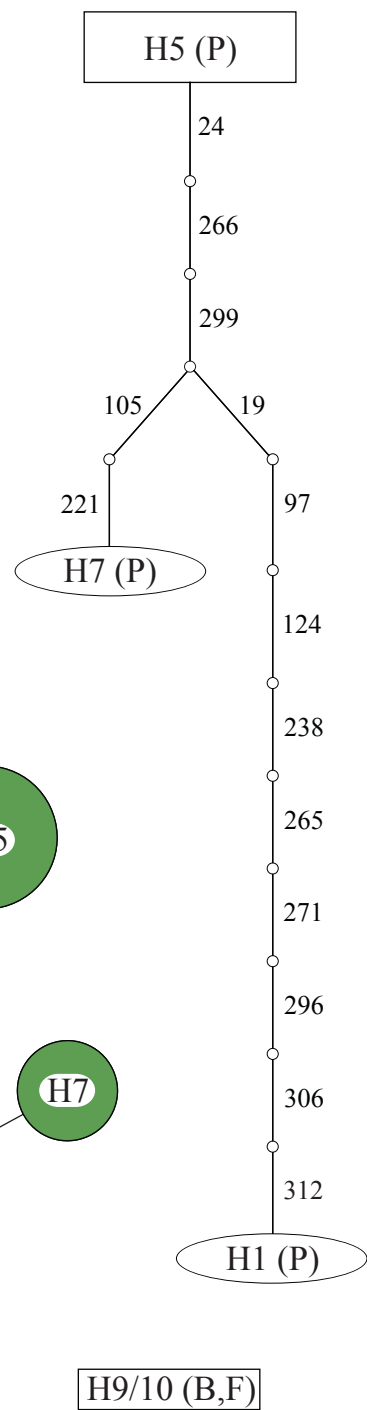

(c)

Supplement: Additional file 2 — Genetic variation based on 9 non-recombining ATP6 sequences of Sebacina epigaea. Haplotypes (H) are defined from ITS + 5.8S + D1/D2 dataset. (a) Maximum likelihood phylogenetic tree. The tree topology was computed from 1000 runs and midpoint rooted. Bootstrap supports (>50%) are shown for each node. Substrate types for the basidiomata are mapped on the topology. eL1 and eL3 represent main lineages. (b) Median-joining network. Circle sizes are proportional to haplotype frequency and connecting lines are proportional to mutation events between haplotypes (numbers of mutated positions are given for all except one mutation). Colours indicate geographical areas where the basidiomata were collected. (c) Statistical parsimony network. Parsimony probabilities were set at 95%. Sizes of circular and rectangular areas are proportional to the number of individuals with that haplotype. Ectomycorrhizal tree families co-occurring in sampling sites are abbreviated as follows: B = Betulaceae, F = Fagaceae, P = Pinaceae. [file 1471-2148-13-102-S2.pdf]

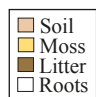

0.02

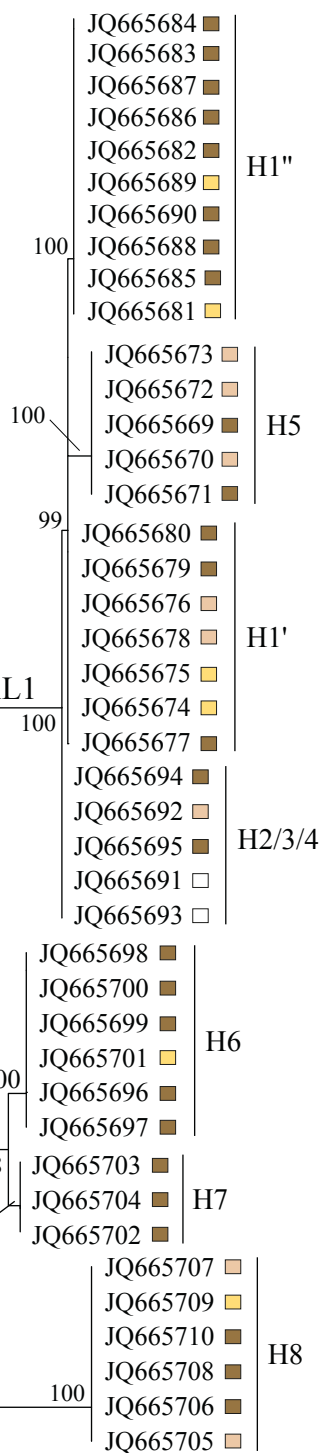

(a)

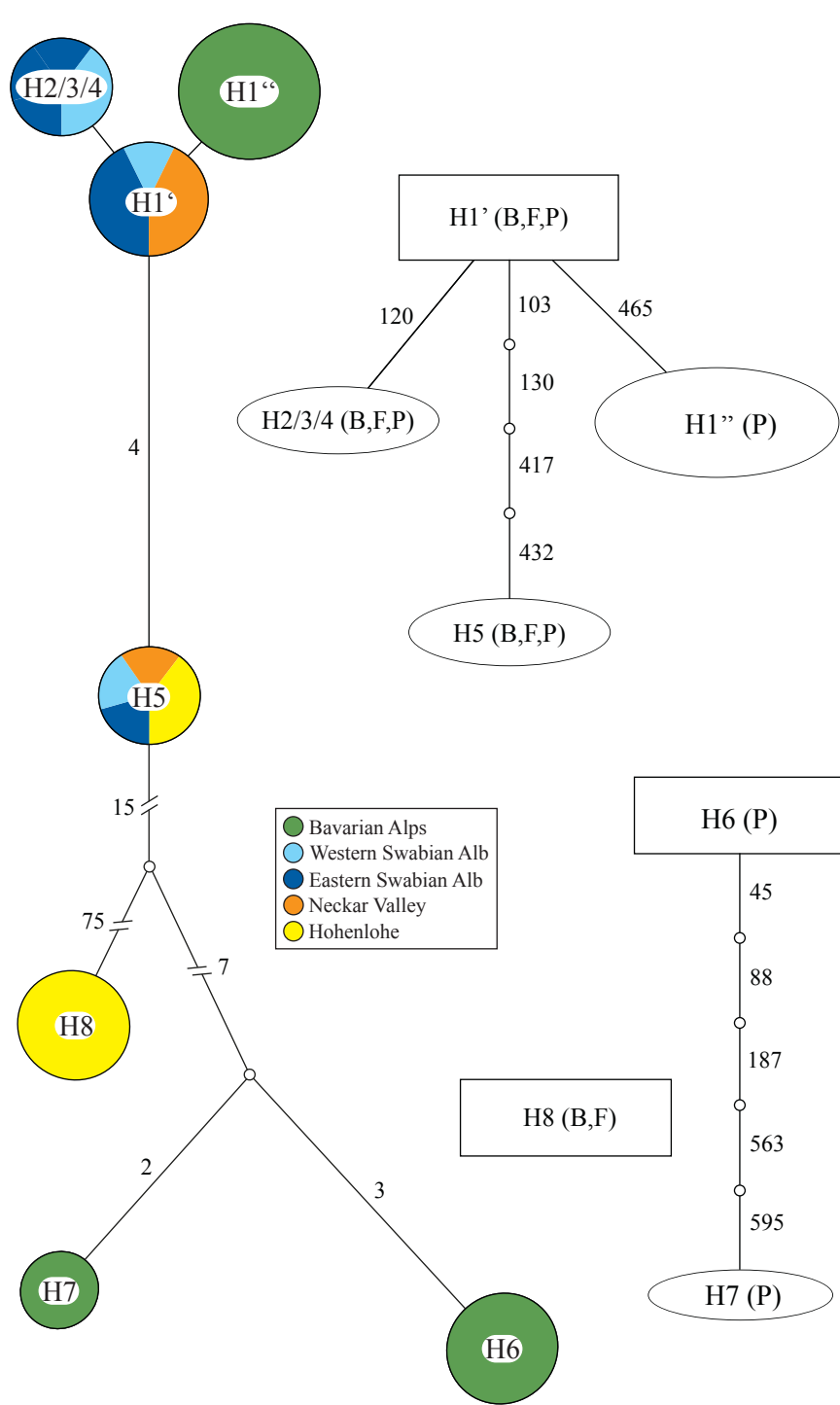

(b)

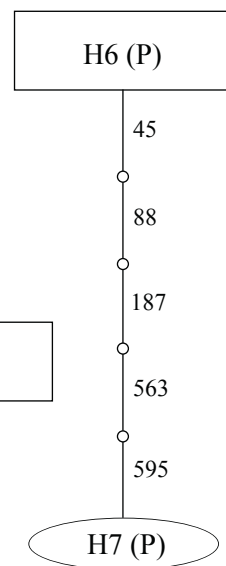

(c)

Supplement: Additional file 4 — Genetic variation based on 42 non-recombining ATP6 sequences of Sebacina incrustans. (a) Haplotypes (H) are defined from ITS + 5.8S + D1/D2 dataset and additional haplotypes found in ATP6 are encoded with apostrophes. Maximum likelihood phylogenetic tree. The tree topology was computed from 1000 runs and midpoint rooted. Bootstrap supports (>50%) are shown for each node. Substrate types for the basidiomata are mapped on the topology. iL1 to iL3 represent main lineages. (b) Median-joining network. Circle sizes are proportional to haplotype frequency and connecting lines are proportional to mutation events between haplotypes (numbers of mutated positions are given for all except one mutation). Colours indicate geographical areas where the basidiomata were collected. (c) Statistical parsimony network. Parsimony probabilities were set at 95%. Sizes of circular and rectangular areas are proportional to the number of individuals with that haplotype. Ectomycorrhizal tree families co-occurring in sampling sites are abbreviated as follows: B = Betulaceae, F = Fagaceae, P = Pinaceae. [file 1471-2148-13-102-S4.pdf]
