# Supplementary material for: High genetic diversity at the regional scale and possible speciation in Sebacina epigaea and S. incrustans
Source: BMC Evol Biol. 2013 May 22;13:102. doi: 10.1186/1471-2148-13-102 (PMC3665632; doi:10.1186/1471-2148-13-102)
Supplement: Additional file 3 — Genetic variation based on 78 non-recombining RPB2 sequences of Sebacina incrustans. Haplotypes (H) are defined from ITS + 5.8S + D1/D2 dataset, additional haplotypes found in the RPB2 are encoded with letters and heterozygous sequences are coded with a and b. (a) Maximum likelihood phylogenetic tree. The tree topology was computed from 1000 runs and was midpoint rooted. Bootstrap supports (>50%) are shown for each node. Substrate types for the basidiomata are mapped on the topology. iL1 to iL3 represent main lineages. (b) Median-joining network. Circle sizes are proportional to haplotype frequency and connecting lines are proportional to mutation events between haplotypes (numbers of mutated positions are given except for all one mutation). Colours indicate geographical areas where the basidiomata were collected. (c) Statistical parsimony network. Parsimony probabilities were set at 95%. Sizes of circular and rectangular areas are proportional to the number of individuals with that haplotype. Distributions of ectomycorrhizal tree families co-occurring in sampling sites are abbreviated as follows: B = Betulaceae, F = Fagaceae, P = Pinaceae. [file 1471-2148-13-102-S3.pdf]

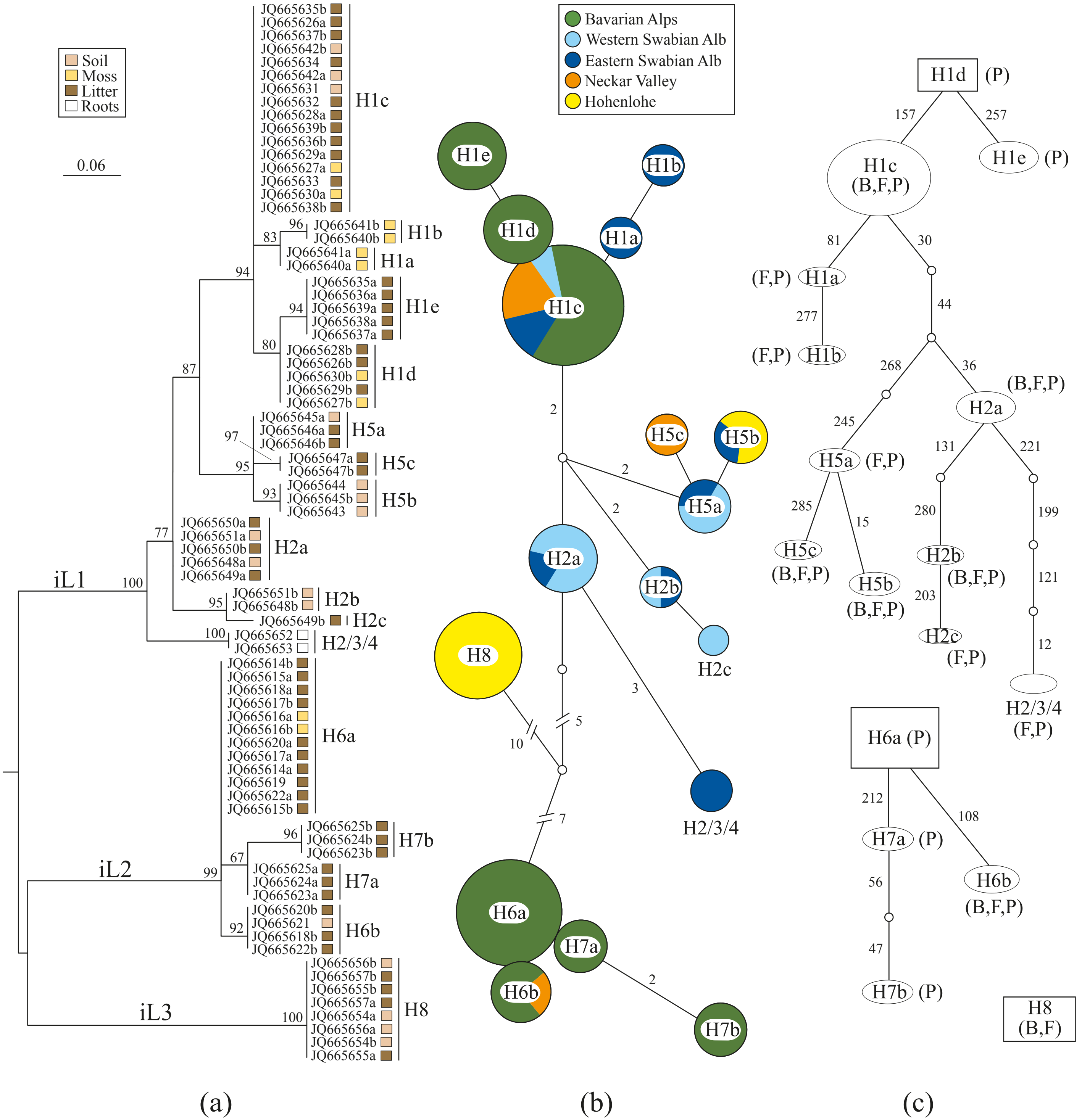

Soil
Moss
Litter
Roots
0.06
JQ665635b
JQ665626a
JQ665637b
JQ665642b
JQ665634
JQ665642a
JQ665631
JQ665632
JQ665628a
JQ665639b
JQ665636b
JQ665629a
JQ665627a
JQ665633
JQ665630a
JQ665638b
H1c
96
83
JQ665641b
JQ665640b
H1b
JQ665641a
JQ665640a
H1a
94
JQ665635a
JQ665636a
JQ665639a
JQ665638a
JQ665637a
H1e
80
JQ665628b
JQ665626b
JQ665630b
JQ665629b
JQ665627b
H1d
87
JQ665645a
JQ665646a
JQ665646b
H5a
97
95
JQ665647a
JQ665647b
H5c
93
JQ665644
JQ665645b
JQ665643
H5b
77
JQ665650a
JQ665651a
JQ665650b
JQ665648a
JQ665649a
H2a
iL1
100
95
JQ665651b
JQ665648b
H2b
JQ665649b
H2c
100
JQ665652
JQ665653
H2/3/4
JQ665614b
JQ665615a
JQ665618a
JQ665617b
JQ665616a
JQ665616b
JQ665620a
JQ665617a
JQ665614a
JQ665619
JQ665622a
JQ665615b
H6a
96
JQ665625b
JQ665624b
JQ665623b
H7b
67
iL2
99
JQ665625a
JQ665624a
JQ665623a
H7a
92
JQ665620b
JQ665621
JQ665618b
JQ665622b
H6b
JQ665656b
JQ665657b
JQ665655b
JQ665657a
iL3
100
JQ665654a
JQ665656a
JQ665654b
JQ665655a
H8
Bavarian Alps
Western Swabian Alb
Eastern Swabian Alb
Neckar Valley
Hohenlohe
H1e
H1b
H1d
H1a
H1c
2
H5c
H5b
2
H5a
2
H2a
H2b
H2c
3
H8
5
10
H2/3/4
7
H6a
H7a
H6b
2
H7b
H1d (P)
157
257
H1c (B,F,P)
H1e (P)
81
30
(F,P) H1a
277
44
(F,P) H1b
268
36
(B,F,P)
H2a
245
H5a (F,P)
131
221
285
15
280
199
H5c
(B,F,P)
H2b
(B,F,P)
H5b
(B,F,P)
203
121
H2c
(F,P)
12
H2/3/4
(F,P)
H6a (P)
212
108
H7a (P)
56
H6b
(B,F,P)
47
H7b (P)
H8
(B,F)
(a)
(b)
(c)
