# Supplementary material for: High genetic diversity at the regional scale and possible speciation in Sebacina epigaea and S. incrustans
Source: BMC Evol Biol. 2013 May 22;13:102. doi: 10.1186/1471-2148-13-102 (PMC3665632; doi:10.1186/1471-2148-13-102)
Supplement: Additional file 5 — Genetic variation of ITS + 5.8S + D1/D2 haplotype sequences inferred from complete (Rec +) and after (Rec -) removing recombination blocks for Sebacina epigaea and S. incrustans datasets. Genetic divergence represents uncorrected p-distances using Mesquite 2.75 [59]. Numbers of unconnected networks based on parsimony networks with a 95% connection probability limit using TCS [53]. n+n = number of dikaryotic samples, n = number of haplotype sequences. [file 1471-2148-13-102-S5.pdf]

	n+n	n	Genetic divergence [%]		No. of haplotypes		No. of unconnected networks	
			Rec +	Rec -	Rec +	Rec -	Rec +	Rec -
S. epigaea								
**eL1**	48	60	7.57	5.16	11	8	7	1
**eL2**	1	2	0.19	0.56	1	2	1	1
**eL3**	1	1	0.00	0.00	1	1	1	1
**All**	50	63	12.11	11.61	13	11	9	3
S. incrustans								
**iL1**	30	44	2.82	1.28	4	5	3	1
**iL2**	12	13	2.72	1.94	2	2	2	1
**iL3**	6	6	0.00	0.00	1	1	1	1
**All**	48	63	7.70	6.25	7	8	6	3
