# Supplementary material for: High genetic diversity at the regional scale and possible speciation in Sebacina epigaea and S. incrustans
Source: BMC Evol Biol. 2013 May 22;13:102. doi: 10.1186/1471-2148-13-102 (PMC3665632; doi:10.1186/1471-2148-13-102)
Supplement: Additional file 7 — Descriptive part of major phylogenetic lineages detected within Sebacina epigaea and S. incrustans. [file 1471-2148-13-102-S7.pdf]

Description of major lineages: Details of the morphology, ecology and geographic distribution of taxa belonging to the genus *Sebacina* analyzed in our paper are provided below. Based on comparative morphological and DNA-sequence data we have characterized the major lineages (those where sufficient populations were sampled) as follows: (1) *Sebacina epigaea* lineage 1 (eL1; haplotypes 1-8); and (2) *S. incrustans* lineage 1 (iL1; haplotypes 1-5), lineage 2 (iL2; haplotypes 6-7) and lineage 3 (iL3; haplotype 8).

(1) *Sebacina epigaea* (Berk. & Broome) Bourdot & Galzin morphospecies

Lineage 1 (eL1; haplotypes 1-8): Basidiomata up to 2 mm thick, resupinate, ceraceous-shiny, gelatinous, merulioid, fresh grey-white and opalescent when fresh. Dried specimens become membranous and grey-brownish in colour. Basidiospores 10–12 × 6–8 µm, Q = 1.33–1.85, mean = 1.67 and S = ± 0.13, ellipsoid, thin-walled, smooth, hyaline. Germinating and resting spores with protuberances present. Phragmobasidia 18–25 × 10–12 (–14) µm, 4-spored, sterigmata 2–3–(4) µm wide. Hyphae of the subhymenium 2.5–3–(4) µm wide, cylindrical, hyaline. Hyphae of the trama 1.5–2.5–(4.0) µm wide, cylindrical, thin-walled, hyaline. Clamp connections absent.

Ecology and distribution: Members of this lineage occur mostly in pure stands of *Picea abies* and less frequently in mixed forests composed of *P. abies* and other tree species (*Abies alba*, *Fagus sylvatica*, *Pinus sylvestris* or *Salix appendiculata*). Only one population sample is from a deciduous forest (*Carpinus betulus*, *Corylus avellana*, *Fagus sylvatica*, *Quercus robur*), see Additional file 6. In most cases, the basidiomata grow directly on naked soil and less frequent on *Picea* litter (Fig. 3) In the public databases, there are some records of *S. epigaea* from Estonia and France. In addition, in Europe several studies have linked this taxon

to ectomycorrhizae of a wide range of plant families (Betulaceae, Cyperaceae, Fagaceae, Malvaceae, Pinaceae, Polygonaceae, Rosaceae and Salicaceae) (Additional file 6).

Comments: There is some variation in the thickness and texture of the basidiomata: thin basidiomata were slightly translucent and smooth and thick basidiomata were opaque and slightly merulioid. However, there were not morphological differences linked to some of the divergent lineages found in *S. epigaea* morphospecies. *Sebacina epigaea* eL1 is one of the most frequent species in *Picea abies* forests. The intraspecific genetic variation in ITS, 5.8S and D1/D2 regions of the nuclear rDNA inferred from a total of 48 population samples ranged from identical to 7.57% based on complete sequences and from identical to 5.16% after excluding recombination blocks. Results from our phylogenetic analyses support the monophyly (100% bootstrap value) of *S. epigaea* eL1 only after excluding recombination blocks.

(2) *Sebacina incrustans* (Pers. ex Fr.) Tul. morphospecies

Lineage 1 (iL1; haplotypes 1-5): Basidiomata up to 3 mm thick, resupinate, cartilaginous, smooth, dirty white to cream. Dried specimens coriaceous and become yellowish. Basidiospores 12–15 × 8–10 µm, Q = 1.33–1.75, mean = 1.57 and S = ± 0.11, ellipsoid to subglobose, thin-walled, smooth, hyaline. Germinating spores with secondary spores present. Phragmobasidia 20–30–(35) × 13–15 µm, 4-spored, sterigmata 2.5–4 µm wide. Hyphae of the subhymenium 3–4 □m wide, cylindrical, hyaline. Hyphae of the trama (2.5)–3.0–4.0 µm wide, cylindrical, thick-walled, up 1 µm, hyaline. Clamp connections absent.

Ecology and distribution: In the sampled sites, collections were found in pure stands of *Picea abies* or mixed forest of *P. abies* and other tree species (*Abies alba*, *Carpinus betulus*,

Corylus avellana, *Fagus sylvatica*, *Larix decidua*, *Pinus sylvestris* and *Quercus robur*). Members of *S. incrustans* iL1 mostly form basidiomata on litter or sometimes on naked soil, above-ground roots or moss (Additional file 6). In addition, population samples belonging to this lineage also have been recorded from Austria, Denmark and Germany. Moreover, representatives of this lineage form ectomycorrhizae with *Kobresia myosuroides*, *Picea abies*, *Bistorta vivipara*, *Populus tremula* and *Salix herbacea* (Austria), *Fagus sylvatica* (Germany) and *Quercus robur* (Italy) (Fig. 2).

Comments: Although members of *S. incrustans* morphospecies exhibit high levels of phenotypic plasticity, they can be easily recognized in the field: with few exceptions, when specimens were growing on naked soil, they were similar in habit and colour to *S. epigaea* morphospecies. Macroscopically, populations within *S. incrustans* iL1 are closely related to and difficult to distinguish from populations of lineages iL2 and iL3. Microscopically, populations of iL1 differ by thick-walled trama hyphae; and iL3 by slightly smaller basidia and basidiospores. Our population samples and sequences in the public databases from Austria, Denmark and Germany of *S. incrustans* formed a distinctive monophyletic group (iL1). Comparative analyses of the ITS, 5.8S and D1/D2 sequences from 30 population samples of iL1 showed genetic variation that ranged from identical to 2.82% based on complete datasets, and from identical to 1.28% after excluding recombination blocks. Furthermore, phylogenetic analyses inferred from complete datasets and those without recombination, supported the monophyly of *S. incrustans* iL1 with significant bootstrap values.

Lineage 2 (iL2; haplotypes 6-7): Basidiomata up to 3 mm thick, resupinate, smooth, cartilaginous, dirty white to cream. Dried specimens become coriaceous and yellowish. Basidiospores 10–14 × 7–9 µm, Q = 1.18–1.75, mean = 1.41 and S = ± 0.14, ellipsoid to

subglobose, thin-walled, smooth, hyaline. Germinating spores with secondary spores present. Phragmobasidia (16–) 18–25 × 13–14 µm, 4-spored, sterigmata 2.5–3 µm wide. Hyphae of the subhymenium 3–4 µm wide, cylindrical, hyaline. Hyphae of the trama (2–) 2.5–4 µm wide, cylindrical, thin-walled, hyaline. Clamp connections absent.

Ecology and distribution: In the sites sampled, most of the collections were found in pure stands of *Picea abies* on moss or litter and only one population was collected from soil in a mixed forest with *Corylus avellana*, *Fagus sylvatica* and *Picea abies* (Additional file 6). So far, a population of this lineage has been reported from ectomycorrhizae with Betulaceae in France (Fig. 2)

Comments: Morphologically, *S. incrustans* iL3 is very similar to iL1 but differs by the thin-walled hyphae of the trama and slightly smaller spores. Sequence variation of ITS, 5.8S and D1/D2 regions of the nuclear rDNA from 12 population samples ranged from identical to 2.72% based on complete sequences, and from identical to 1.94% after eliminating recombination blocks. Phylogenies inferred from complete datasets and those without recombination supported the monophyly of *S. incrustans* iL2.

Lineage 3 (iL3; haplotype 8): Basidiomata up to 3 mm thick, resupinate, smooth, cartilaginous, dirty white. Dried specimens become coriaceous and yellowish to ochraceous. Basidiospores 10–13 × 6.5–8.0 µm, Q = 1.25–2.0, mean = 1.63 and S = ± 0.16, ellipsoid to subglobose, thin-walled, smooth, hyaline. Germinating spores present. Phragmobasidia (13)–15–20 × 10–11 µm, 4-spored, sterigmata 2–3 µm wide. Hyphae of the subhymenium 2–3.5 µm wide, cylindrical, hyaline. Hyphae of the trama (2–) 2.5–4 µm wide, cylindrical, thin-walled, hyaline. Clamp connections absent.

Ecology and distribution: Specimens belonging to this lineage were found directly on soil, litter of *Fagus sylvatica* or on moss. So far, populations of *S. incrustans* iL3 are known only from one site in Germany in a frondose forest composed of *Carpinus betulus*, *Corylus avellana*, *Fagus sylvativa* and *Quercus robur* (Additional file 6). Furthermore, the ITS sequences of this lineage proved to be identical to three collections identified as *S. incrustans*, which were collected from a *Picea* plantation in Estonia. Also some *S. incrustans* iL3 form ectomycorrhiza with *Quercus petraea* in France (Fig. 2).

Comments: Microscopically, the slightly smaller basidia and basidiospores can be used to separate *S. incrustans* iL1 from iL3. Populations of iL3 had identical ITS, 5.8S and D1/D2 sequences and formed a monophyletic group in phylogenies inferred from complete datasets and those without recombination.
