# Supplementary material for: High genetic diversity at the regional scale and possible speciation in Sebacina epigaea and S. incrustans
Source: BMC Evol Biol. 2013 May 22;13:102. doi: 10.1186/1471-2148-13-102 (PMC3665632; doi:10.1186/1471-2148-13-102)
Supplement: Additional file 8 — List of primers and their nucleotide sequence used in this study. For primer references see [60-64]. Primers marked with asterisk are only used for DNA sequencing. [file 1471-2148-13-102-S8.pdf]

Marker name	Primer sequence 5'→3'	Reference
**(a) ITS+D1/D2**		
ITS1F	CTTGGTCATTTAGAGGAAGTAA	[60]
NL4	GGTCCGTGTTTCAAGACGG	[61]
ITS4*	TCCTCCGCTTATTGATATGC	[62]
LR0R*	ACCCGCTGAACTTAAGC	R. Vilgalys lab: biology.duke.edu/fungi/mycolab/primers.htm
5.8SR*	TCGATGAAGAACGCAGCG	[63]
**(b) RPB2**		
fRPB2-5F	GAYGAYMGWGATCAYTTYGG	[64]
bRPB2-7.1R	CCCATRGCYTGYTTMCCCATDGC	[25]
sRPB2-5.1F	CTGCTAYAGAARAAGG	This study
sRPB2-7R	ATGATGCTYGAACAAC	This study
fRPB2-6F*	TGGGGYATGGTNTGYCCYGC	[25]
sRPB2-6.5F*	TCTTCGTAGTTGATGG	This study
sRPB2-6.1R*	AGRTTCTTGACAAGCC	This study
**(c) ATP6**		
ATP6-3	TCTCCTTTAGAACAATTTGA	[26]
ATP6-4	AAGTACGAAWACWTGWGMTTG	[26]
sATP6-3	TGAAGTYGTAAGTCTTTTAGG	This study
